# Orbital angular momentum–driven multistate photomemory

**DOI:** 10.1126/sciadv.adx8795

**Published:** 2025-10-10

**Authors:** Ye-Ru Chen, Po-Wen Wang, Wen-Hao Chang, Jagdees Prasad, Yu-Yang Chen, Yu-Cheng Chiu, Ting-Hua Lu, Yann-Wen Lan

**Affiliations:** ^1^ Department of Physics, National Taiwan Normal University, Taipei 116, Taiwan.; ^2^ Department of Chemical Engineering, National Taiwan University of Science and Technology, Taipei 116, Taiwan.

## Abstract

Current optical memory technologies face critical challenges, including limited precision in multistate control, energy inefficiency, and inadequate adaptability across diverse material platforms. To directly address these issues, this study introduces a noncontact approach by exploiting the unique properties of the orbital angular momentum (OAM) of light. The distinctive longitudinal electric field generated by OAM light substantially enhances the density of trap states in materials such as molybdenum disulfide, as the primary material for investigation, and others as supplementary examples. This enhancement enables precise modulation of key device characteristics, including readout current, hysteresis window, and charge storage capacity, with quantitative behavior accurately modeled by the Poole-Frenkel effect. Our results reveal the transformative potential of OAM light in enabling multilevel memory states with exceptional tunability and versatility across different material systems. This work underscores the viability of OAM-driven memory as a platform for the next generation of highly functional, optically responsive memory devices.

## Teaser

A degree of freedom, orbital angular momentum of light, optically and noninvasively controls multistate photomemory in MoS_2_.

## INTRODUCTION

In recent years, the need for advanced optoelectronic memory devices that can achieve high on/off current ratios, extended retention times, and multilevel storage capabilities has intensified (*1*–*4*), especially with the push toward miniaturized, wearable, and flexible electronics (*5*, *6*). Optical control has emerged as a powerful approach for these applications, offering noncontact, low-power operation that reduces wear on materials and increases device longevity (*7*, *8*). Light-driven methods provide unique advantages for creating highly responsive, energy-efficient memory systems, marking an essential step forward in the development of atomic-scale, multifunctional, and sustainable memory devices (*9*, *10*).

Atomically thin monolayer MoS_2_, an outstanding candidate for two-dimensional semiconducting transition metal dichalcogenide (2D TMDC) material with a hexagonal van der Waals structure, has garnered a lot of attention. Research on multilayer MoS_2_ shows that bulk MoS_2_ has an indirect bandgap of ~1.29 eV, transitioning to a direct bandgap of ~1.8 eV in monolayers, thereby enhancing light-matter interactions (*11*–*14*). This makes monolayer MoS_2_ an ideal channel material for field-effect transistors (FETs) with high mobility (200 cm^2^ V^−1^ s^−1^) and an on/off current ratio of ~10^8^, enabling applications in FETs (*15*–*17*), light-emitting diodes (*18*–*20*), and solar cells (*21*, *22*). 2D TMDCs have high thermal stability, mechanical flexibility, optical transparency, a large surface-to-volume ratio, low power consumption, atomic-scale thickness, adjustable energy bandgap, unique crystal structures, and reasonable carrier mobility along the basal plane (*23*). Because of outstanding photoresponse and excellent immunity to short-channel effects, monolayer MoS_2_ is currently believed to offer considerable potential for a variety of future photonic and optoelectronic memory devices (*15*, *24*, *25*). Three-terminal optoelectronic memory devices, including charge-trapping memory, floating gate memory, ionic liquid transistors, gate-tunable memory, and memtransistors, were developed applying TMDCs because of their high density and variable charge transfer capabilities (*26*). The photocurrent and photoresponse times of nonvolatile memory devices are changed by the trapping/detrapping of electrons and holes in the charge storage media (*27*, *28*). These earlier studies showed that achieving outstanding and reliable nonvolatile memory features requires careful selection of charge-trapping materials and modification of their nanostructure (*29*, *30*).

In the optoelectronic memory field, crystalline silicon remains the most widely used channel material. However, the physical thickness of the silicon layer limits the effective carrier mobility, posing challenges for device scaling and the reduction of short-channel effects. In addition, the charge-trapping phenomenon, which plays a crucial role in many fundamental physics problems, holds great potential in driving the development of innovative synaptic devices and accelerating the commercialization of 2D electronic devices.

An orbital angular momentum (OAM) of light, or so-called twisted light (TL), also known as the physics of optical vortices, is a light property that is associated with the spiral phase pattern of a wavefront. The winding level of a twisted wavefront is proportional to $e^{i\ell \phi }$ in the transverse plane of its field profile. Each photon of TL has a momentum with the magnitude of ℓℏ, where ℓ is any integral number named as topological charge (*31*, *32*). Through its tunable quantum number ℓ , the photon’s OAM offers an additional and wider degree of freedom to customize light-matter interaction. Many research areas have benefited from the use of TL interaction with condensed matter, including quantum entanglement, biomedicine, optical communication systems, optical manipulation, and imaging systems (*33*, *34*). Because of its essential characteristics in optical communication for information storage, management, and transportation, the OAM of light has garnered a lot of interest now. OAM of light has many applications in optical data transmission, high-capacity spatial mode division multiplexing, in-memory computing, nanoscale optical tweezers, high-dimensional quantum information storage and transmission, and integrated photonics. It can influence optoelectronic memory devices by modulating the optical and electrical properties of matter (*35*–*38*). This has various essential implications for both long- and short-range optical communication and quantum information processing (*38*).

The OAM of light interaction with 2D materials, such as monolayer MoS_2,_ for atomic-scale memory devices and how it affects memory behavior has still not been studied. The future forecasts will inevitably include some unexpected and anticipated findings that might be studied considering the OAM of light interaction with monolayer MoS_2_ and their potential uses in next-generation optoelectronics and integrated photonic circuits (*34*). We have initiated quite a bit of research work focused on controlling and modulating the unique and unprecedented electrical and optical properties of monolayer MoS_2_ using distinct OAM of light-caused variations in the *z*-direction electric field of a nonvolatile optoelectronic memory device. By manipulating its topological charge ℓ , it can offer an additional and wider degree of freedom to store, control, and transport information in light-matter interaction. To date, an inferiority of research has been directed toward studying the OAM of light beam interaction with the monolayer MoS_2_ mechanism.

Here, we demonstrated that a large area of continuous monolayer MoS_2_ was transferred on a silicon substrate covered with a thin layer of thermal oxide SiO_2_ by a chemical vapor deposition (CVD) technique. We report the development of nonvolatile optoelectronics on an effective charge-trap memory transistor at the MoS_2_/SiO_2_ dielectric interface, where the linear dependence of incident OAM of light power was systematically studied. Our findings show that the high-performance photocurrent, hysteresis window, and charge storage performance of monolayer MoS_2_ memory devices are effectively controlled and modulated by different OAM of light. The MoS_2_ optical memory characteristic is dependent on exposure time, measurement temperature, and OAM of light intensity. Using the OAM of light, our study presents a groundbreaking noncontact, low-power method for achieving multistate memory control in 2D materials. This light-driven approach not only aligns with the demands of sustainable, energy-efficient computing but also enables stable multistate memory effects with potential applications in nonvolatile memory technology. By leveraging optical control for precise, multistate functionality, this research offers a transformative pathway toward next-generation memory devices that could revolutionize data storage and green computing.

## RESULTS

### Structure and electrical properties of the functional trap MoS_2_ device

**Fig. 1.**
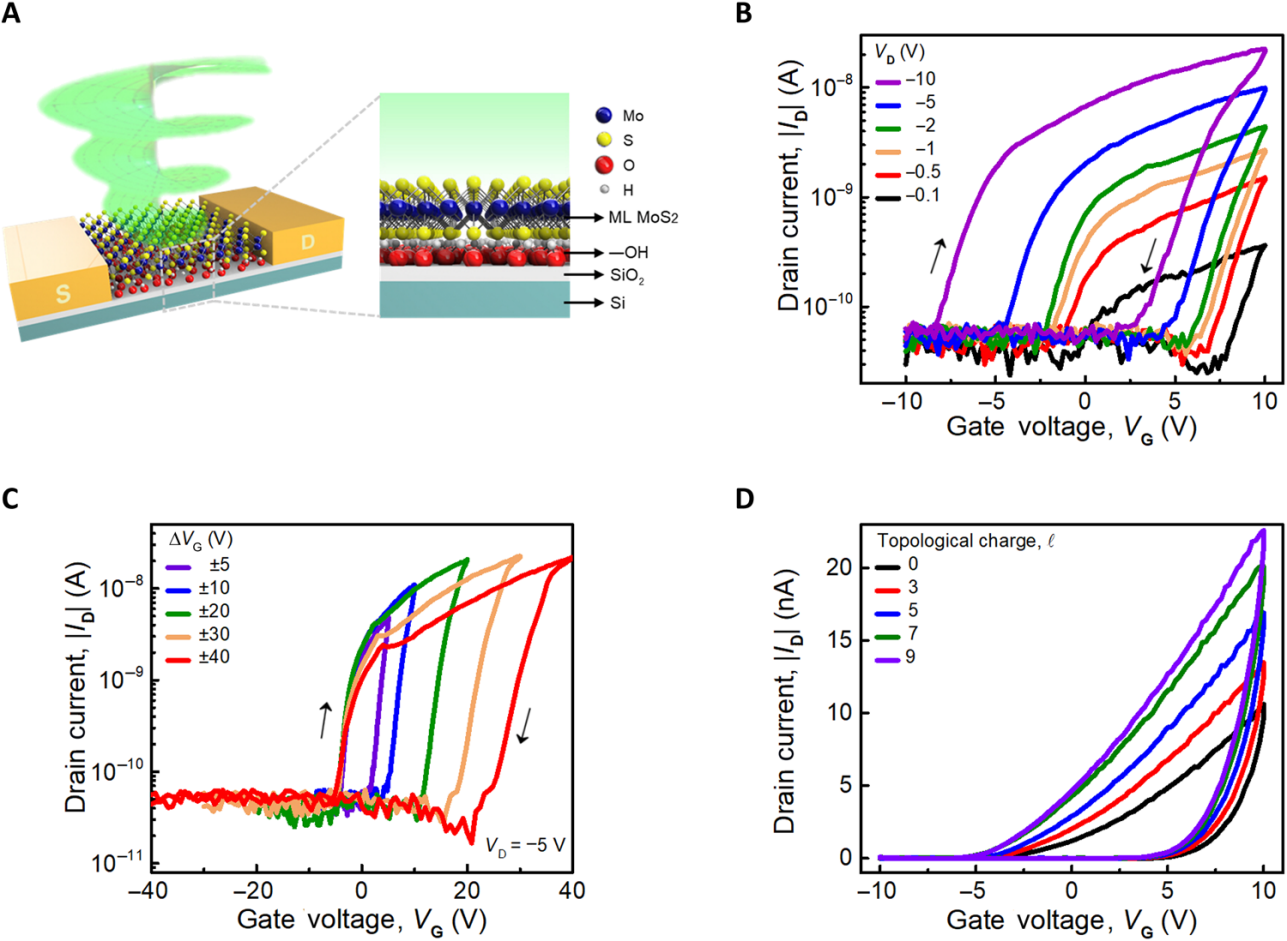
Structure schematic and electrical properties of functional trap MoS_2_ device with/without OAM light. (**A**) Sketch of the device structure and illustration of OAM light shining on the functional trap on the substrate. ML, monolayer. (**B**) Transfer characteristics of a functional trap MoS_2_ device without light illumination. The black arrows show the measurement direction. (**C**) Transfer characteristics are shown by different gate voltage measurement ranges without light illumination. (**D**) The transfer characteristic after irradiation by the OAM of light.

Figure 1A schematically depicts the complete device structure that is under irradiation by the OAM of light (the experimental setup is described in section S1, and the device optical microscopy image is provided in fig. S2A). To demonstrate that trap state can be controlled by light, the dielectric SiO_2_ substrate on a highly doped silicon substrate and a vast area of atomically thin MoS_2_ layers intentionally enclosed a hydroxyl layer (─OH functionalized). To improve the ability of the hydroxyl layer, we applied surface chemistry–based treatment (O_2_ plasma method) on the SiO_2_ substrate before wet transferring the MoS_2_ layer (the photoluminescence and Raman characteristics of the MoS_2_ layer are provided in fig. S2, B and C) (*15*). This was achieved by producing several ─OH functional group trap states on the SiO_2_ surface, which served as artificial charge trap states for a memory device. The performance of 2D MoS_2_ memory devices can be altered through surface treatment with O_2_ plasma to vary the current density, charge transfer, molecule polarities, and interfacial phononic characteristics. Compared to an untreated O_2_ plasma on a SiO_2_ substrate (fig. S2, D and E), these functional trap states can further enhance the MoS_2_ memory transistors’ optical programmability and longer retention time.

For a memory device, the hysteresis window refers to the range of voltage or current values over which the device exhibits nonlinear behavior in response to input signals, for instance, the applied gate voltage ( $V_{G}$ ). Usually, bistable or multistable states exist in the device, which are responsible for this nonlinear behavior since they retain information long after the input signal is gone. The $I_{D}$ values were measured with $V_{G}$ sweeping from −10 to +10 V and back to −10 V with various bias voltages ( $V_{D}$ ) under dark conditions (in Fig. 1B). This indicates that the $V_{D}$ can modulate the device’s hysteresis window and that when a greater bias voltage is supplied, a broader clockwise hysteresis window is obtained (the $I_{D}$-$V_{D}$ characteristic is presented in fig. S3A). The arrows indicate the forward and backward sweeping, respectively. The $V_{D}$ drives the charge carriers to flow through the channel, which improves the rate of carriers captured by the trap states in the forward-sweeping region and then forms a localized electric field. The localized field serves as an effective gate field that rapidly reduces the carrier concentration and cuts off the channel that switching the device back to the off-state earlier in the backward-sweeping region. The higher magnitude of $V_{D}$ is applied to the device to be opened earlier while sweeping $V_{G}$ from −10 to 10 V. However, because of the trapped charges at the interface, the device turns off quickly in the backward-sweeping region and then indicates the broadened hysteresis window. On the other hand, we present the results using different $V_{G}$ sweeping ranges with a fixed $V_{D}$ of −5 V, in which the $V_{G}$ measurement range is varied in order of ±5 to ±40 V, as shown in Fig. 1C. For the memory properties of MoS_2_ transistors, the concentration of electrons in the monolayer MoS_2_ is modulated by applying positive or negative gate voltages and is easily trapped by the SiO_2_ surfaces. The ─OH functional group of SiO_2_ has the charge-trapping property, which traps the photogenerated hole carriers, hence facilitating the photoresponse on MoS_2_. The photogating effect reveals an apparent rise in current because of a shift in the threshold voltage ( $V_{\text{TH}}$ ), which is caused by the trapped charges changing the effective gate voltage (*33*). No matter which starting $V_{G}$ is used, it demonstrates that the device opens at a similar region of $V_{G}$ in the forward region. This is consistent with the findings in Fig. 1B, which show that the opening region is controlled by the $V_{D}$ . In the backward-sweeping region, the device rapidly turns off and displays a broader hysteresis window. The stability of data storage is guaranteed by the larger memory window. Typically, the gate voltage regulates the carriers’ storage and release within charge traps. This kind of functional trap device is used to control memory states by the OAM of light in this study. Figure 1D demonstrates a controllable device hysteresis window programmed by the OAM light. The details about OAM light modulation on MoS_2_ memory transistors are discussed in the following.

### OAM light–induced modulation of hysteresis and readout current

**Fig. 2.**
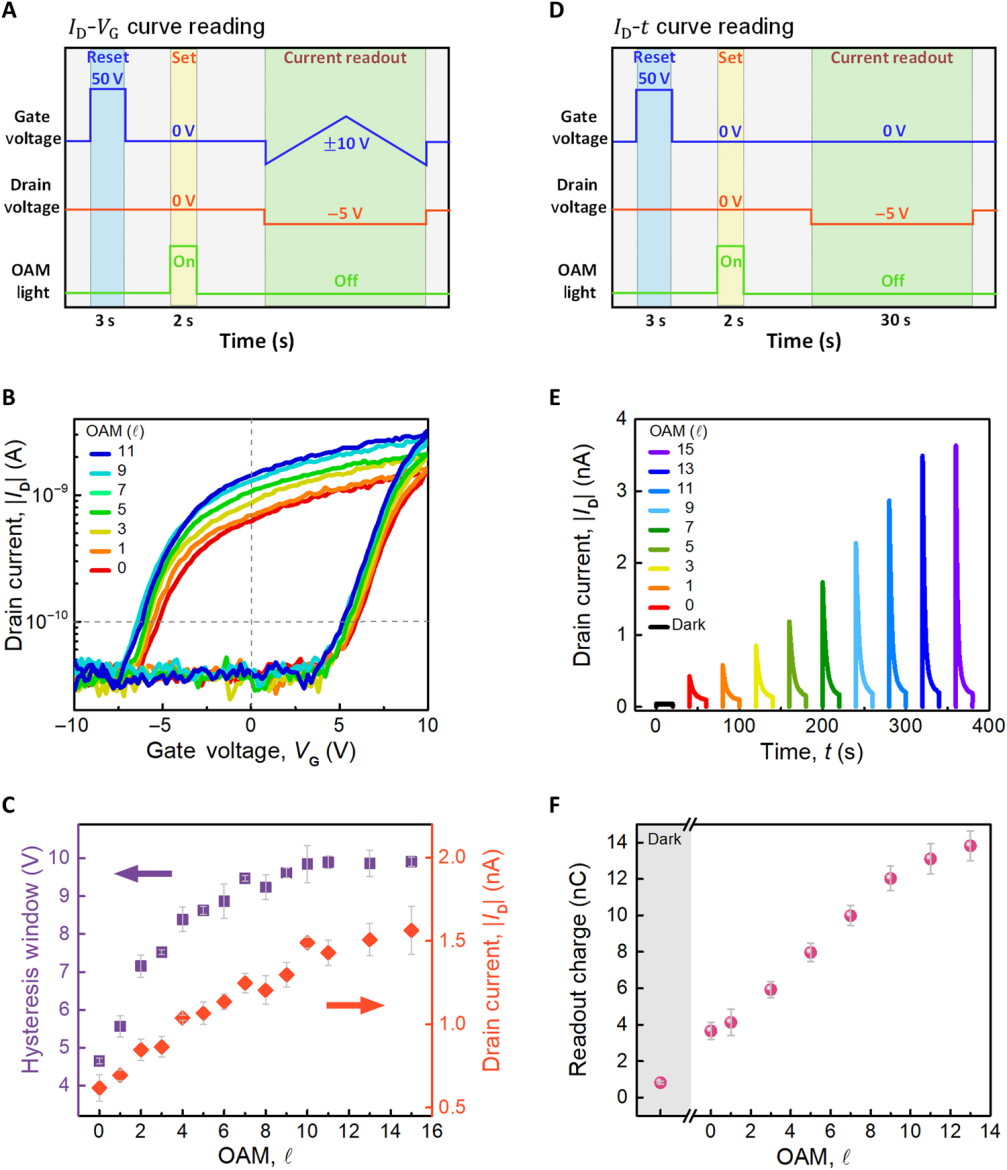
The electrical property modulation by exposure to light with OAM. (**A** and **D**) Experimental procedure shows the experiment step with the reading of $I_{D}\;‐\;V_{G}$ and $I_{D}\;‐\;t$ measurements. The reset process is given by gate voltage, and the set process is given by laser exposure. (**B**) $I_{D}\;‐\;V_{G}$ readout with $V_{G}$ change from −10 to +10 V by different OAM of light exposure**.** (**C**) Readout current and hysteresis window extract by $I_{D}\;‐\;V_{G}$ curve. (**E**) $I_{D}\;‐\;t$ readout by different OAM light exposure. $V_{G}$ is fixed at 0 V and $V_{D}$ is 5 V. (**F**) Readout charge read by $I_{D}\;‐\;t\;$ curve.

To explore the influence of OAM of light on MoS_2_ memory devices, we construct two different operating sequences to observe the readout procedure of the device after the illumination of light under various orders of OAM. Figure 2 (A and D) demonstrates the measuring procedures, where both of them use a 50-V $V_{G}$ pulse with a time duration of around 3 s as a reset procedure and then set the device by irradiating the OAM of light with a time duration of 2 s for the subsequent experiments. (The examination results of the pulse amplitude are provided in fig. S3, B and C.) The difference between the two procedures is the readout method: One uses the $V_{G}$ hysteresis sweeping within the range of ±10 V, with a step of 0.2 V and a fixed $V_{D}$ of −5 V, to define the hysteresis window; and the other one takes the constant $V_{D}$ at −5 V applying for 30 s without $V_{G}$ and records the result of $I_{D}\;‐\;\text{t}$ to extract the readout charges. The $V_{G}$ hysteresis sweeping results, with the measuring delay of 100 ms, as shown in Fig. 2B, reveal that the hysteresis window gradually increases, as the higher order of OAM is applied. The hysteresis window and the readout current are extracted at the fixed $I_{D}=0.1\;\text{nA}$ and $V_{G}=0\;$ V, respectively, where the hysteresis loops under conditions of OAM-carrying light have been measured three times. Figure 2C demonstrates the analyzed hysteresis window (purple) and the readout current (red) versus the order of OAM, ℓ . The results reveal that the OAM of light enhances both the hysteresis window and readout current of the device as the rising order of ℓ but saturates in the region with a higher ℓ . It is caused by the exceeding irradiation region of the device, while the illumination area with the rising ℓ is expanding. On the other hand, the readout current is also conducted by the $I_{D}\;‐\;\text{t}$ measurement, depicted in Fig. 2E, which provides a direct observation of the readout charge by the integration of the curve. The increase of the $I_{D}$ values can also be observed with the rising ℓ. Figure 2F illustrates the extracted readout charge from the $I_{D}\;‐\;\text{t}$ measurement, where the vertical axis represents the reading charge obtained by integrating over 30 s in the curve. This can be regarded as the differences in the quantities of written charge caused by different OAM of light. The “Dark” value corresponds to the black line in Fig. 2E, indicating the readout without writing by laser. When considering the increase in OAM order, the corresponding expansion of the beam’s irradiation area can contribute to a higher number of photogenerated carriers trapped. However, through experiments examining the effect of exposure area, we found that light carrying OAM induces additional effects beyond solely increasing the irradiation area, directly associated with the presence of OAM, such as OAM-modified local electric field distributions. The influence of varying exposure area through OAM light is discussed in detail in section S4.

### Power, exposure time, and temperature dependence of OAM light effects on memory devices

**Fig. 3.**
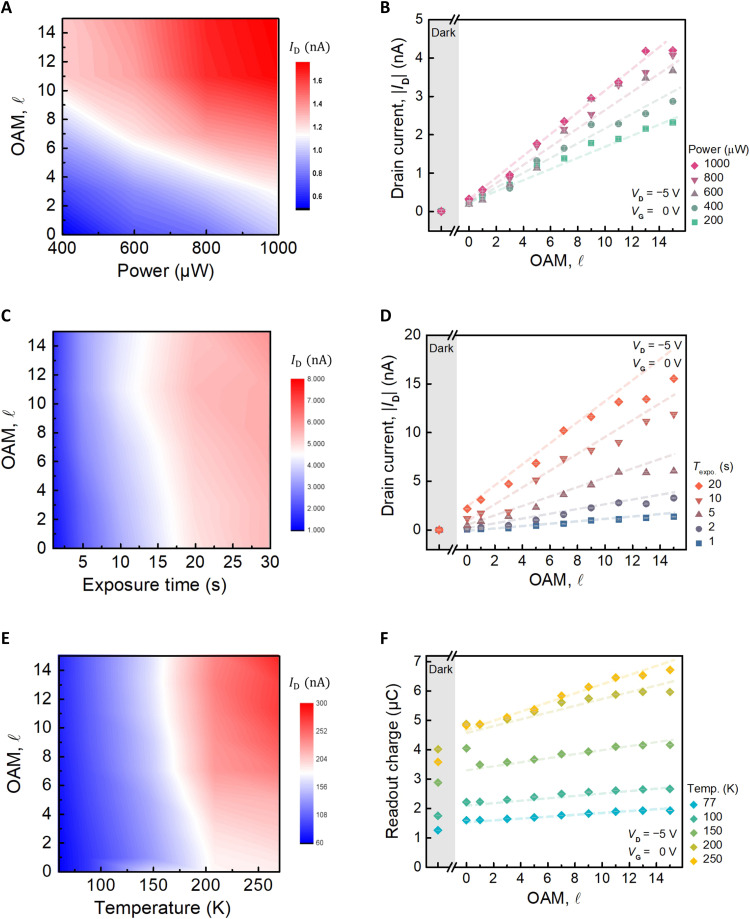
The impact of OAM light on MoS_2_ memory devices under power, exposure time, and temperature. (**A**) $I_{D}\;‐\;V_{G}$ curve readout current control by the power of light and OAM of light. (**B**) $I_{D}\;‐\;t$ curve readout current control by the power of light and OAM. (**C**) $I_{D}\;‐\;V_{G}$ curve readout current control by exposure time and OAM of light. (**D**) $I_{D}\;‐\;t$ curve readout current control by exposure time and OAM. (**E**) $I_{D}\;‐\;V_{G}$ curve readout current control by the temperature and OAM of light. (**F**) $I_{D}\;‐\;t$ curve readout current control by the temperature and OAM.

To investigate the impact of OAM of light on memory devices under various physical conditions. We first characterize the power dependence of the incident light during the optical writing process. In practice, the laser passes through an optical attenuator to adjust its intensity. A light intensity detector senses and records the required light intensity at the sample side. The power of the incident laser ranges from 200 to 1000 μW , as depicted in Fig. 3 (A and B), which present the readout results from $I_{D}\;‐\;V_{G}$ and $I_{D}\;‐\;t$ measurements, respectively. The results demonstrate that light intensity also gradually enhances the readout current. The white region in Fig. 3A highlights the advantage of using OAM light, where higher orders of OAM enable comparable or even enhanced device performance in low-power regions, relative to simply increasing optical power. This demonstrates the improved energy efficiency imparted by the OAM of light, allowing the device to operate effectively with reduced power consumption. Second, we varied the exposure time during the optical writing process by controlling the electronic shutter to generate light pulses with various exposure durations, as depicted in Fig. 3C. The results demonstrate the readout by $I_{D}\;‐\;V_{G}$ with the exposure time ranging from 0.6 to 30 s, where the horizontal axis represents the exposure time and the vertical axis represents the OAM order ( ℓ ). The current readout values ( $I_{D}$ ) represent the current extracted from the $I_{D}\;‐\;V_{G}$ curve while passing through $V_{G}=0\;V$ during forward sweeping. Again, the white area shows that the higher OAM of light requires a lower exposure time to achieve comparable readout charges. Figure 3D illustrates the $I_{D}\;‐\;t$ readout results, where the horizontal axis represents the order of ℓ and the vertical axis represents the extracted current of the $I_{D}\;‐\;t$ curve, in which the colors represent the results of various exposure times [fig. S5 (A and B) indicates that the $I_{D}\;‐\;t$ curve refer to Fig. 3 (B and D)]. It is observed that apart from changing the exposure time, which substantially affects the readout of the memory device, the OAM, as a physical degree of freedom of light, can independently control the readout current. Both exposure time and OAM of light are proportionally correlated with the current readout and charge readout. Last, we conduct the temperature-dependent measurements. The sample was placed into a variable-temperature system chamber and cooled using liquid nitrogen in a vacuum environment. Measurements are taken as the temperature changes, with a waiting period of 30 min after each temperature adjustment to ensure thermal equilibrium. The temperature ranges from 77 to 250 K. Figure 3 (E and F) illustrates the $I_{D}\;‐\;V_{G}$ and $I_{D}\;‐\;t$ readout results under various temperatures, respectively. The device hysteresis loops under atmosphere/vacuum conditions and the readout current/hysteresis window under various temperatures set by fundamental light are presented in section S6. The result shows that the readout current apparently increases in the vacuum when compared to atmospheric conditions. This is attributed to eliminating gas interference, such as water vapor from the environment, resulting in purer and more stable electronic properties of MoS_2_. Moreover, both the readout current and readout charge decrease as the temperature decreases. This indicates that the operation mechanism of charge traps involves thermal effects. When the temperature decreases, the thermal agitation limits the movement of carriers between charge traps, leading to a decrease in both readout current and readout charge. The white area in Fig. 3E also emphasizes that the relatively low-power operation occurs in the cases of OAM light at lower temperatures. On the other hand, since higher-order OAM beams have larger ring diameters, the electric field maximum may lie closer to the electrodes, potentially leading to local modulation of the transition metal dichalcogenide (TMD) doping and the Schottky barrier. However, our temperature-dependent analysis (*39*) indicates that these spatially induced local gating effects do not notably affect the effective Schottky barrier height. This confirms that the observed memory effects are not primarily governed by local variations at the metal-semiconductor interface (see section S7 for details).

### OAM light–induced multistate memory characterization

**Fig. 4.**
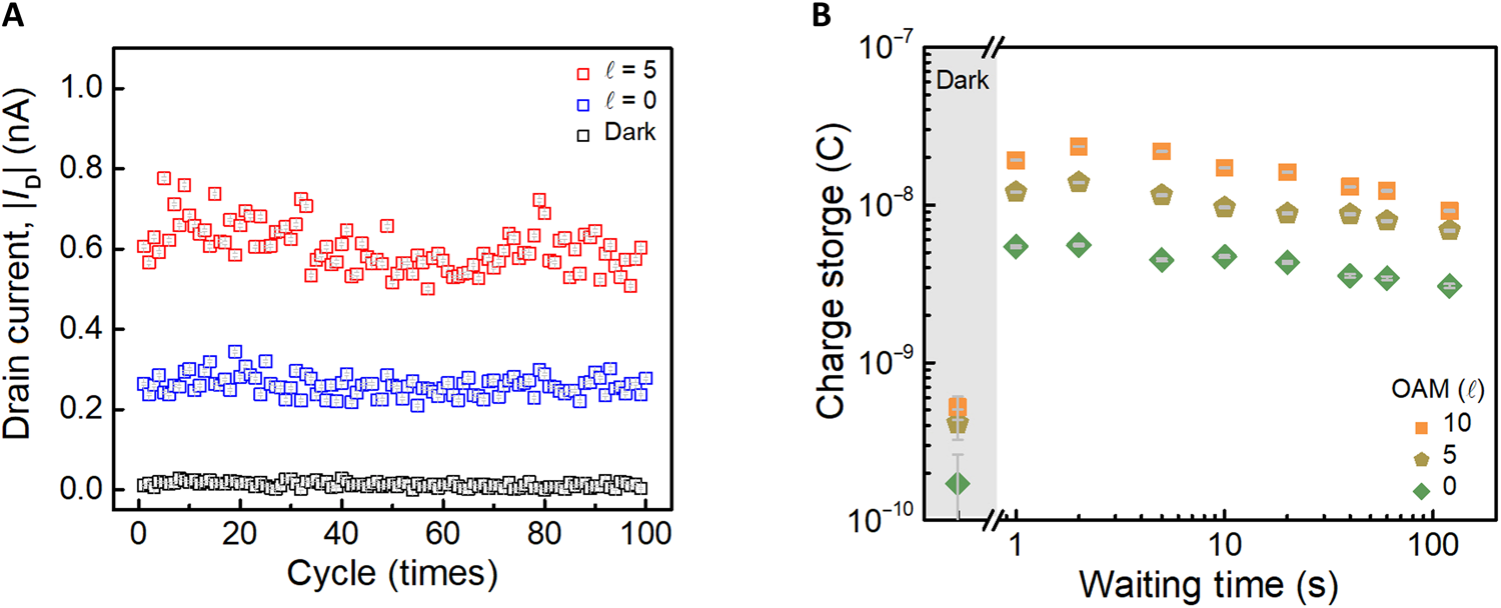
Multiple memory-state characterization of functional trap MoS_2_ device controlled by OAM of light. (**A**) Endurance characteristics of the memory device. The program process is set by fundamental light and OAM light, with the OAM equal to 5, respectively. The erase process is reset by a positive gate voltage pulse and the readout by sweeping $V_{G}$ from −10 to 10 V and then back to −10 V with a fixed $V_{D}$ of −5 V. The cycling process is repeated 100 times. (**B**) The waiting time test for the memory device’s storage ability. Charge storage read by $I_{D}\;‐\;t$ curve after a long time of waiting.

To examine the memory characteristics of memory devices modulated by OAM of light, we present the storage capabilities of this memory device. Figure 4 illustrates the endurance and retention time of the memory under atmospheric pressure and room temperature conditions. In Fig. 4A, the repeatability and durability of the device characteristics are tested through repeated erase-write-read cycles. The vertical axis represents the readout current, while the horizontal axis represents the number of repeated reads. Each reading cycle consists of the following steps: (i) direct measurement of the readout results after erasure without optical writing, (ii) the erasure followed by optical writing with a light intensity of 600 μW for 2 s and then readout, and (iii) the erasure followed by an optical writing with a light intensity of 600 μW and the order of OAM ℓ=5 for 2 s and then readout. The readout procedure is the same as the previous, sweeps $V_{G}$ from −10 to 10 V and then back to −10 V with a fixed $V_{D}$ of −5 V, and extracts the $I_{D}$ while $V_{G}=0$ V. In Fig. 4A, the black, blue, and red squares represent the results of readout at dark conditions, ℓ=0 , and ℓ=5 , respectively. The cycle is repeated 100 times, confirming that the light-stored memory functionality remains reliable after multiple cycles. To further verify the endurance, we conducted extended cycling tests on another device, which completed 1000 programming cycles, and the results are included in section S8. These highlight the repeatability and durability of the multistate memory behavior enabled by OAM light–based programming of the devices. Figure 4B illustrates the memory retention time of the device, testing its ability to retain charge storage after optical writing. This depends on whether the charge traps effectively capture carriers and prevent the recombination of charge carriers between the trap layer and the channel. The method involves an erasing procedure using positive gate voltage, followed by optical writing with the OAM, ℓ = 0, 5, and 10, with a fixed light intensity and exposure time of 600 μW and 2 s, respectively. After writing, the light source and bias are turned off, followed by a waiting period before a readout procedure. The horizontal axis represents different wait times ranging from 1 to 120 s, while the vertical axis represents the charge readout value obtained from the $I_{D}\;‐\;t$ curve. The result shows that the device can maintain its memory even after a power off and can be read out after a certain period. The device retention is also examined and provided in section S8. The device can maintain the written state from different OAM lights for up to 100 s and is distinguished by the different order of OAM.

## DISCUSSION

### OAM-induced longitudinal fields for trap density modulation

The photogating effect indicates that incident light strikes a material, which triggers transitions between energy bands and induces transitions within trap states. These trap states can arise from an oxygen plasma–treated SiO_2_ substrate, which creates hydroxyl group trap states at the interface between the channel and the dielectric layer. When light irradiates, electrons or holes can be trapped at these trap states, forming a localized gate voltage and modulating the carrier concentration in the device channel. This leads to a horizontal shift in the transfer characteristic curve ( $I_{D}\;‐\;V_{G}$ ) due to the change in threshold voltage ( $V_{\text{TH}}$ ), which denotes the threshold that turns the device into an on state. In the case of our study, holes are trapped, and the concentration of free electrons in the conduction band increases, causing a negative shift in the transfer curve and broadening the hysteresis window after illumination with light. The unique wavefront structure characterized by OAM light exhibits the electromagnetic field perpendicular to its propagation direction and additional components in the *z* direction. In a study investigating the optical chirality of OAM light, it is introduced the existence of a longitudinal electric field ( $E_{z}$ ) from a vortex light (*40*). The representation of the electric field for a plane wave under the Laguerre-Gaussian (LG) profile is

$$E\left(r,t\right)=E_{0}\left(\alpha \hat{x}+\beta \hat{y}\right)f_{\text{LG}}$$ (1)

where $E_{0}$ represents the amplitude of the electric field, α and β are its components in the *x* and *y* directions, and $f_{\text{LG}}$ denotes the LG field profile

$$\begin{array}{c} \begin{array}{c} f_{\text{LG}}=\sqrt{\frac{2p!}{\pi \omega_{0}^{2}\left(p\;+|\ell |\right)!}}\frac{\omega_{0}}{\omega \left(z\right)}{\left[\frac{\sqrt{2}r}{\omega \left(z\right)}\right]}^{|\ell |}\\ L_{p}^{|\ell |}\left[\frac{2r^{2}}{\omega^{2}\left(z\right)}\right]e^{\left[\frac{-r^{2}}{\omega^{2}\left(z\right)}\right]}\times e^{i\left[kz+\ell \;\phi -\omega t+\frac{kr^{2}}{2R\left(z\right)}-\left(2p+|\ell |+1\right)G\left(z\right)\right]} \end{array} \end{array}$$ (2)

in which $\omega_{0}$ , ω , R , k , $L_{p}^{|\ell |}$ , and G denote the beam waist, beam radius, radius of curvature, wave number, Laguerre polynomial, and Gouy phase, respectively. The study proposed that, on the basis of the original *x*-*y* plane components of the electric field, once the OAM of light is generated, a correction term corresponding to the *z* direction should be added, which is expressed as

$$\begin{array}{c} \begin{array}{c} E\left(r,t\right)=\\ E_{0}\left\{\;\left(\alpha \hat{x}\;+\;\beta \hat{y}\right)+\hat{z}\frac{i}{k}\left[\alpha \left(\;\text{γcos}\phi \;-\;\frac{i\ell }{r}\text{sin}\phi \right)+\beta \left(\;\text{γsin}\phi \;+\;\frac{i\ell }{r}\text{cos}\phi \right)\right]\right\}f_{\text{LG}} \end{array} \end{array}$$ (3)

From above, we can observe the field distribution in the *z* direction corresponding to different radii r and azimuthal angles ϕ in the transverse plane.

**Fig. 5.**
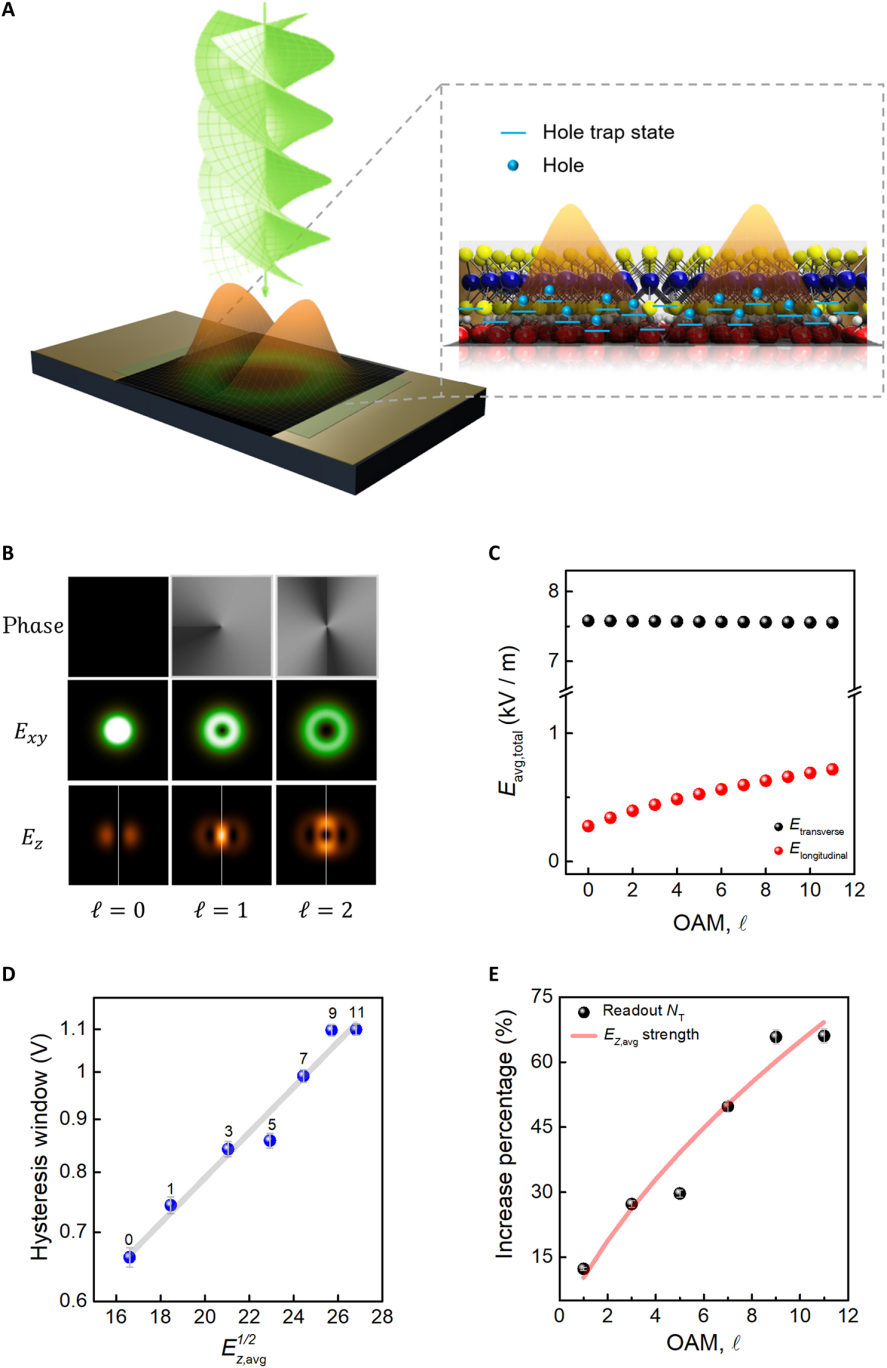
Mechanism discussion of the functional trap MoS_2_ device. (**A**) The sketch of an incident OAM of light that generates the *z*-direction electric field and its distribution. The zoomed-in figure demonstrates the induction of trapped holes formed by $E_{z}$ . (**B**) The nine squares present the simulated results of the phase, transverse field, and longitudinal field distributions. (**C**) The estimated area-averaged electric field strength versus the OAM, ℓ . Black and red dots indicate the field strength from the transverse and longitudinal components, respectively. (**D**) The hysteresis window under logarithmic scale versus the square root of longitudinal field strength indicates a linear dependence, in which numbers above the blue dots represent the OAM, ℓ , and the gray line depicts the linear fitting result. (**E**) The increased percentage when compared to the fundamental Gaussian light. The red line indicates the simulated $E_{z,\text{avg}}$ strength as the change of the order of OAM  $\left[\left(E_{z,\text{avg}}^{\ell }-E_{z,\text{avg}}^{\ell =0}\right)/E_{z,\text{avg}}^{\ell =0}\right]$ . The black dots present the estimated trap density from the readout charges  $\left[\left(N_{T}^{\ell }-N_{T}^{\ell =0}\right)/N_{T}^{\ell =0}\right]$.

The γ  $\left[\gamma =\left(\frac{|\ell |}{r}-\frac{2r}{\omega^{2}}+\frac{\mathrm{ikr}}{R}-\frac{4r}{\omega^{2}}\frac{L_{p-1}^{|\ell |+1}}{L_{p}^{|\ell |}}\right)\right]$  is a coefficient that reflects the change by the OAM order, ℓ . For a plane wave, whose electromagnetic field oscillates in the *x*-*y* plane and propagates in the *z* direction; however, OAM light results in variations of the electric field in the *z* direction ( $E_{z}$ ), thereby altering the interaction between light and semiconductor materials, as depicted in Fig. 5A. The orange region represents the $E_{z}$ generated by the OAM light that is assumed to arise from the twisted wavefront, which is distinct from the former planar wavefront. In the field profile, we use the linearly polarized light ( α=1;β=0 ) and isolate the component of the *z*-direction electric field, which is shown in Fig. 5B. The nine squares demonstrate the phase, intensity distribution of the transverse field ( $E_{x,y}$ ), and longitudinal field ( $E_{z}$ ) distribution for OAM light through numerical calculation, in which ℓ ranges from 0 to 2. It is observed that the intensity distribution of the $E_{z}$ is dependent on the azimuth angle. We infer that, except for the electric field in the *x*-*y* plane, the $E_{z}$ also modulates the photogating effect, increasing the capture of hole traps in the material. As depicted in Fig. 5A, when the OAM of light is normally incident on the surface of the device, the green ring represents the electric field intensity in the *x*-*y* plane, while the orange peak represents the corresponding $E_{z}$ intensity. Accordingly, on the basis of Eq. 2, we estimate the area-averaged electric field strengths  $\left(E_{\text{avg}}=\sqrt{\frac{\int_{A}{|E|}^{2}\mathrm{dA}}{A}}\right)$  of the transverse and longitudinal fields generated by the OAM of light (details of the field strength estimation are provided in section S9). Figure 5C presents the calculated results for the transverse (black) and longitudinal (red) field strengths as functions of the topological charge. Notably, the $E_{z}$ field strength increases noticeably with the magnitude of the OAM, while the transverse component shows a slight decrease. Compared to plane waves, when light carries OAM, there is also a contribution from the longitudinal components, leading to a more pronounced photogating effect in its irradiation region. That is, light with OAM induces more hole trap capture, which improves the performance of the memorial devices. This can be referred to as the Poole-Frenkel effect, which describes a mechanism of electrical conduction in materials, particularly semiconductors, in which an external electric field can lower the energy barriers for carrier emission from localized trap states (*41*). Carriers could be retrapped, which is proportional to the square root of field strength $E^{1/2}$ , depending on the carrier that occurs by the nearest neighboring trap or after a drift (*42*). This suggests that the Poole-Frenkel effect can reflect the threshold voltage shift ( $∆V_{\text{TH}}$ ) under the $I_{D}\;‐\;V_{G}$ sweeping with the changing trap density (*43*, *44*), in which logarithmic $∆V_{\text{TH}}$ linearly depends on the $E^{1/2}$. Figure 5D demonstrates the logarithmic scale of hysteresis window change ( ∆V ) versus the square root of longitudinal field strength $E_{z,\text{avg}}^{1/2}$ from the OAM lights that correspond to the order of OAM ranges from 0 to 11 (as labeled above the blue dots). This result agrees with the Poole-Frenkel effect, in which the $E_{z}$ from OAM lights induces trap density in the programming. To estimate the density of the trapped holes, we further calculate the hole trap density ( $N_{T}$ ) according to the experimental results, where the relation between trap density and hysteresis window shift can be expressed as (*45*, *46*),

$$N_{T}=\frac{C_{\text{ox}}\Delta V}{q}$$ (4)

From above, $N_{T}$ is the trap density, $C_{\text{ox}}$ is the capacitance of the oxide layer, q is the elementary charge, and ΔV is the change in the hysteresis window. The impact of longitudinal fields on the devices is assumed to be proportional to the increase in trap density, as illustrated in Fig. 5E.

The red line represents the increment in $E_{z,\text{avg}}$ strength induced by the OAM of light and the corresponding numerical aperture (NA = 0.25). The black dots depict the calculated trap density based on the readout values obtained from hysteresis windows after writing with different OAM of light at 77 K. The reason we measure at 77 K is to minimize the thermal-induced effects. The increasing percentage is defined and indicates the enhanced rate of $E_{z,\text{avg}}$ and readout trap density when comparing the results under OAM irradiation to the fundamental light, which is denoted by $\left(E_{z,\text{avg}}^{\ell }-E_{z,\text{avg}}^{\ell =0}\right)/E_{z,\text{avg}}^{\ell =0}$ and $\left(N_{T}^{\ell }-N_{T}^{\ell =0}\right)/N_{T}^{\ell =0}$ , respectively. The trend of increment in trap density shows a good agreement with the calculated $E_{z}$ intensity as the rising OAM, ℓ . This result indicates a strong correlation between the increase in trap density and the longitudinal electric field strength contributed by different OAM orders. This demonstrates that the OAM of light holds crucial potential for enabling multilevel memory states in storage devices. The effects of positive and negative OAM orders are provided in section S10. Similar multilevel readout current behavior induced by OAM light is also observed in memory devices based on different material systems, as provided in section S11. To provide a broader context for evaluating our device performance, we have included a comparison table in section S12, summarizing key parameters from previously reported TMD-based photomemory devices (*47*–*51*). While our device may not outperform all existing works in terms of absolute metrics, this study aims to demonstrate the introduction of OAM as an additional degree of freedom for photomemory control. By adjusting the OAM of the light source during the writing process, this additional degree of freedom can be used, offering the possibility of increased information density in future memory applications. Furthermore, it allows for fine-tuning of the device’s performance, opening up opportunities for enhancing the efficiency and versatility of memory technologies.

The OAM of light forms a twisted wavefront, which generates the longitudinal electric field ( $E_{z}$ ), appreciably enhancing the formation of hole trap states in the memory device. This unique field structure, distinct from planar light, effectively modulates the photogating effect, leading to improved control over the electrical properties such as readout current, hysteresis window, and stored charge. Our findings demonstrate that using OAM of light to irradiate MoS_2_-based memory devices introduces an additional degree of operational freedom, enabling multilevel memory states in future optically sensitive memory devices. By using OAM, we introduce a potential pathway for noncontact and energy-efficient control over data storage and logic operations.

## MATERIALS AND METHODS

### Synthesis of MoS_2_

MoS_2_ is synthesized on a sapphire substrate using the CVD method at a temperature of 190°C. In this process, molybdenum trioxide (MoO_3_) powder is placed in a quartz boat at the center of the furnace’s heating zone, with the sapphire substrate positioned adjacent to it. In addition, another quartz boat containing sulfur (S) powder is placed upstream in the furnace. Argon gas flow is used to transport the vapors of both sulfur and MoO_3_ toward the sapphire substrate, enabling the reaction and deposition of MoS_2_ on the substrate during the synthesis process.

### Device fabrication

The sample substrate used in this study consists of a silicon base with a 300-nm-thick silicon dioxide (SiO_2_) layer. A grid-like Ti/Au electrode pattern ( 150μmby150μm with 100-μm spacing) was defined and deposited onto the SiO_2_ layer through electron beam lithography. Following this, the substrate underwent 30W , 60-s oxygen plasma treatment. A large-area monolayer of MoS_2_ is then wet transferred onto the prepared substrate.

The process started with applying a poly(methyl methacrylate) (PMMA) coating to the MoS_2_ layer, spun at 4000 rpm for 55 s. The sapphire/MoS_2_/PMMA assembly is immersed in an ammonia solution for 3 hours to separate the MoS_2_/PMMA film from the sapphire substrate. After transferring the MoS_2_ onto the Si/SiO_2_ substrate, the sample is baked on a hot plate to remove any residual moisture, and the PMMA layer is dissolved using acetone. For device channel etching, photolithography is used. After spin-coating the photoresist, the channel pattern is defined with a glass photomask and ultraviolet exposure. The unwanted areas of MoS_2_ are etched using argon plasma (60 W for 30 s, repeated twice), and the photoresist is lastly removed with acetone to complete the device fabrication.

## Supplementary Materials

### This PDF file includes:

Sections S1 to S12 Figs. S1 to S10 Table S1
