# Supplementary material for: Orbital angular momentum–driven multistate photomemory
Source: Sci Adv. 2025 Oct 10;11(41):eadx8795. doi: 10.1126/sciadv.adx8795 (PMC12513417; doi:10.1126/sciadv.adx8795)
Supplement: Supplementary file 1 — Sections S1 to S12 Figs. S1 to S10 Table S1 [file sciadv.adx8795_sm.pdf]

Science Advances
AAAS

Supplementary Materials for

Orbital angular momentum–driven multistate photomemory

Ye-Ru Chen *et al.*

Corresponding author: Ting-Hua Lu, thlu@ntnu.edu.tw; Yann-Wen Lan, ywlan@ntnu.edu.tw

Sci. Adv. **11**, eadx8795 (2025)
DOI: 10.1126/sciadv.adx8795

This PDF file includes:

Sections S1 to S12
Figs. S1 to S10
Table S1

Section 1. Experimental setup.

The 532 nm laser is used as an excitation source, passing through a half-wave plate (HWP) to tune the polarization that satisfies the conditions for the spatial light modulator (SLM). The computer-generated hologram then projects onto the SLM to transfer the light into the OAM-carried light with the selected order of ℓ. The beam splitter (BS) separates the fundamental and OAM of light, which makes the OAM-carried light toward the object. The device is fabricated using a silicon substrate with a 300 nm dielectric layer (SiO_2) and nine-square electrodes, followed by the oxygen plasmonic treatment to generate the -OH on the surface of SiO_2, which serves as a functional group trap state.

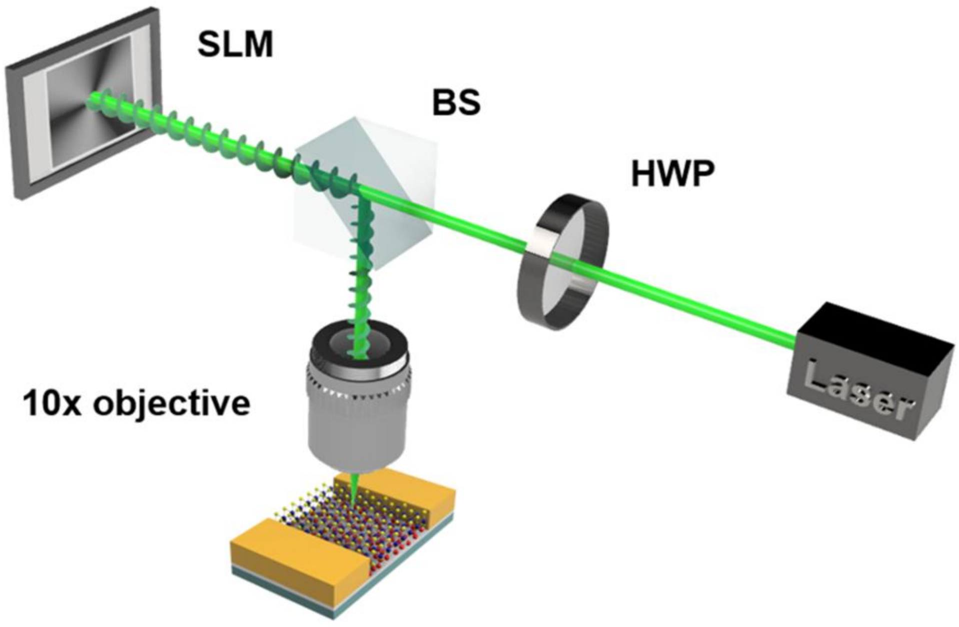

Figure S1. A schematic representation of the complete device structure with optical setup.

Section 2. Device optical characterization and comparison.

Figure S2A provides the optical microscope image of the MoS_2 memory device used in this study. **Figures S2B** and **S2C** display the results of photoluminescence (PL) analysis and Raman spectroscopy performed on the MoS_2 channel between two electrodes after the fabrication process. The MoS_2 exhibits excellent luminescence properties. In **Fig. S2B**, the Raman analysis shows peak values for the E_{2g} and A_{1g} modes at ~385.8 cm^{-1} and ~404.7 cm^{-1}, respectively. The peak difference of approximately ~18.9 cm^{-1} confirms the monolayer structure of the MoS_2 in this device. **Figures 2D** and **2E** demonstrate the OM image and $I_D - V_G$ hysteresis sweeping on a device without surface treatment by O_2 plasma.

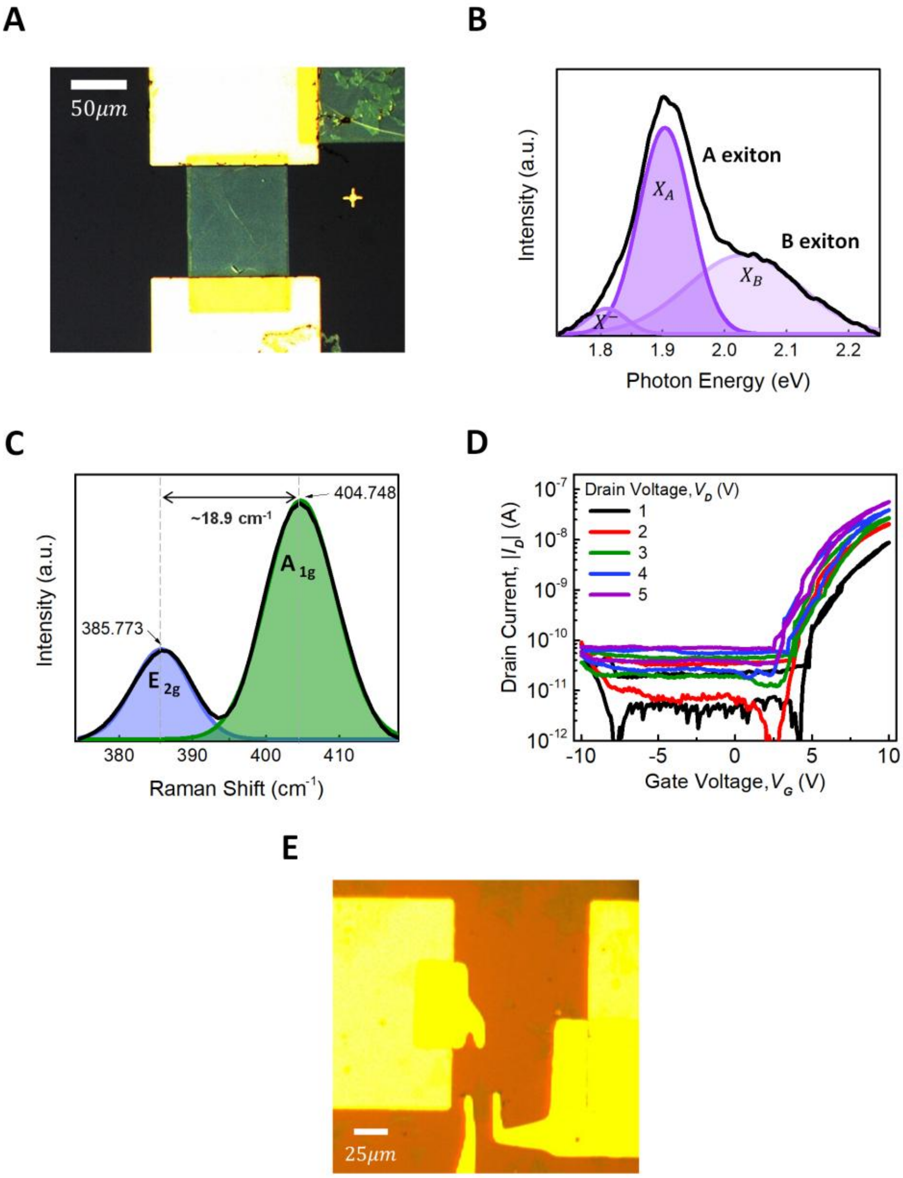

Figure S2. Characterization of MoS_2 memory devices with and without O_2 plasma treatment.
(A) The optical microscope image of the MoS_2 memory device with O_2 plasma treatment. (B) Photoluminescence analysis chart. (C) Raman analysis chart of a monolayer MoS_2 sample. (D) $I_D - V_G$ of the MoS_2 in the dark environment with different V_D ranges. (E) Optical microscope image of MoS_2 memory device without O_2 plasma treatment.

Section 3. Memory device characteristics.

Figure S3A shows the $I_D - V_D$ characteristics of the sample in the dark without any write or erase procedures, with V_G ranging from -15 V to 15 V with increments of 5 V. It can be observed that when $V_D <$ -5 V, the device is in the off-state. In the erase procedure, to ensure that the reset gate voltage effectively returns the sample to a low current state, we conducted tests to evaluate the impact of gate voltage pulse intensity on the erase capability. **Figure S3B** shows the $I_D - V_G$ readout results after writing with a 600 μW, 532 nm laser for 2 seconds, followed by different gate voltage pulse intensities, each applied for 3 seconds. It is evident that as the positive voltage pulse intensity increases, the sample's read current decreases. In **Fig. S3C**, the horizontal axis represents the gate voltage pulse intensity, and the vertical axis shows the read current values for each curve after V_G = 0 V. This indicates that a higher positive pulse intensity results in a better erase effect. Therefore, in the experimental procedure, an erase step typically involves applying a +50 V gate voltage for 3 seconds to ensure complete erasure of the sample.

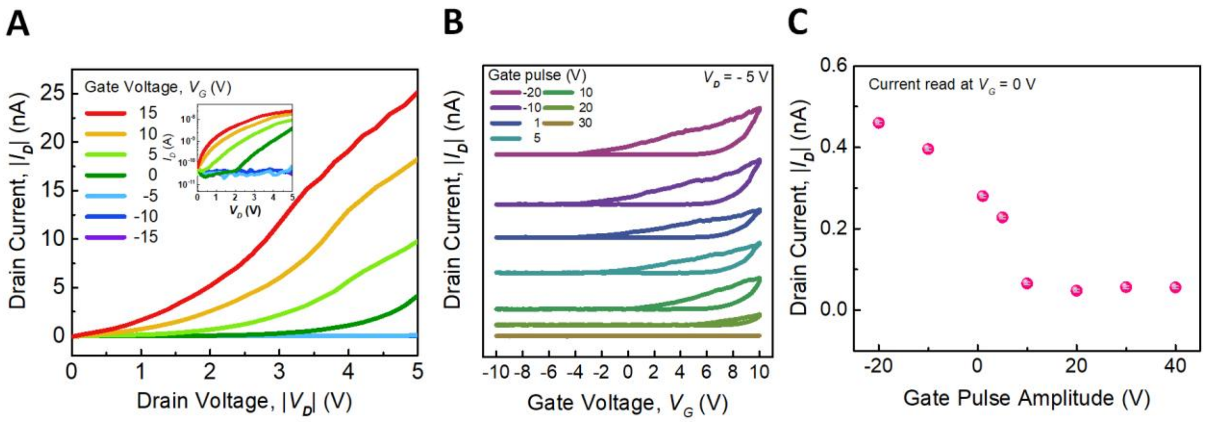

Figure S3. Electrical erasing characteristics and gate pulse dependence.

(A) Relationship of $I_D - V_D$ with different V_G, **(B)** Recycle of $I_D - V_G$ current readout, followed by different gate pulses for erasing. **(C)** Examining the gate pulse amplitude for the reset process.

Section 4. Irradiating area and power density.

To gain a deeper understanding of the impact of orbital angular momentum (OAM) light on the readout of optically sensitive memory, we compared the readout current at V_G = 0 V from the $I_D - V_G$ curve under different irradiation areas. As shown in **Fig.S4A**, the fundamental light represents the laser with ℓ = 0, which carries no OAM. The irradiation area of this fundamental beam was adjusted by defocusing the beam, thereby changing the area it covered. In contrast, OAM beams intrinsically have different irradiation profiles depending on OAM, ℓ, resulting in varying illumination areas.**Fig.S4** illustrates the comparison between the fundamental light and OAM light, in which **Fig.S4A** corresponds to fixed total power intensity and **Fig.S4B** fixed power density, showing the relation between irradiation area and readout current. It is observed that, whether total power intensity or power density is held constant, the OAM beams produce a notably stronger influence on the readout current compared to the fundamental beam. Additionally, we intentionally defocused the beams to vary the irradiation area while keeping the power density constant, meaning that the total laser power was increased proportionally with the expanded beam area. Furthermore, under conditions of constant irradiation area, achieved by varying the OAM order without altering the beam size (**Fig. S4C**), we still observed a clear increase in the device's readout current with increasing OAM. This suggests that the enhancement in memory performance is not solely attributable to the enlarged exposure area. Instead, it points to additional mechanisms intrinsically linked to the presence of OAM, such as OAM-induced modifications to the local electric field distribution. To decouple the role of the OAM phase structure from simple intensity redistribution, we conducted a control experiment using structured light that does not carry OAM. Specifically, we created a donut-shaped intensity pattern using a hologram generated by a spatial light modulator (SLM), carefully designed to replicate the spatial distribution of an $\ell = 1$ beam, as shown in the inset of **Fig. S4D**. In the inset, the top row displays the hologram used to generate the donut-shaped beam and its corresponding OM image (from left to right), while the bottom row shows the case of the OAM beam with $\ell = 1$. After an optical programming process under identical total power intensity, we compared the resulting $I_D - t$ readout curves (**Fig. S4D**). The data clearly show that the $\ell = 1$ beam induces a considerably higher readout current than the non-OAM donut beam. This result highlights the unique role of the helical phase structure of OAM light in modulating the device's memory behavior, likely through enhanced carrier trapping mechanisms that go beyond the effects of intensity distribution alone.

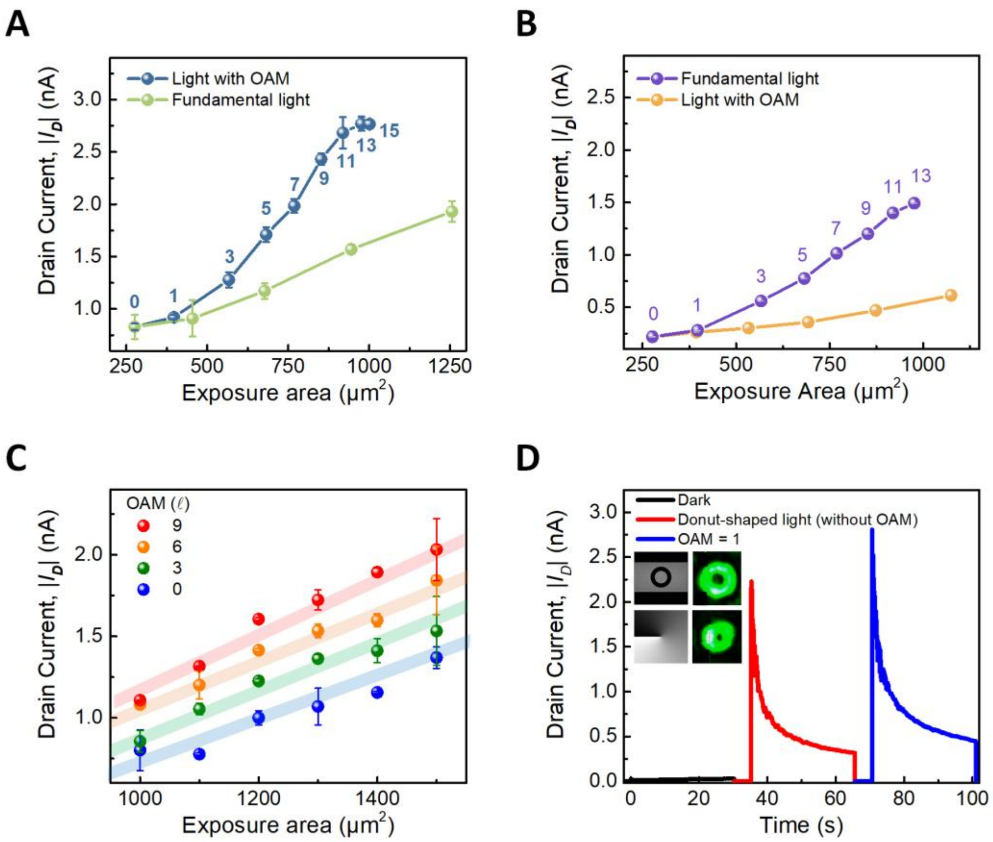

Figure S4. Control experiments examining the influence of power, power density, and irradiation area on OAM-induced effects.

(A) Fixed total power intensity and compare the $I_D - V_G$ current read by OAM light exposure with origin area and fundamental light exposure with a different area. **(B)** The same as **(A)** but fixed the power density. **(C)** Fixed power density and read the $I_D - V_G$ current while controlling the same irradiating area for every condition of light that expose on the device. **(D)** The $I_D - t$ current readout for the comparison of the programming using donut-shaped light (without OAM), and the OAM of light ($\ell = 1$). In the inset, the top row displays the hologram used to generate the donut-shaped beam and its corresponding OM image (from left to right), while the bottom row shows the case of the OAM beam with $\ell = 1$.

Section 5. The $I_D - t$ readout results.

Figures S5A to **S5C** present the $I_D - t$ readout results, which are under the condition of $V_D = -5$ V and $V_G = 0$ V, that refer to **Fig. 3B**, **3D**, and **3F**. All the readout procedures have a time duration of 30 seconds. In **Fig. 3B** and **3D**, the recorded Drain current (I_D) is extracted at $t = 0$ for each readout, refer to **Fig. S5A** and **S5B**. The readout charge shown in **Fig. 3F** is extracted from the integrated result in **Fig. S5C**.

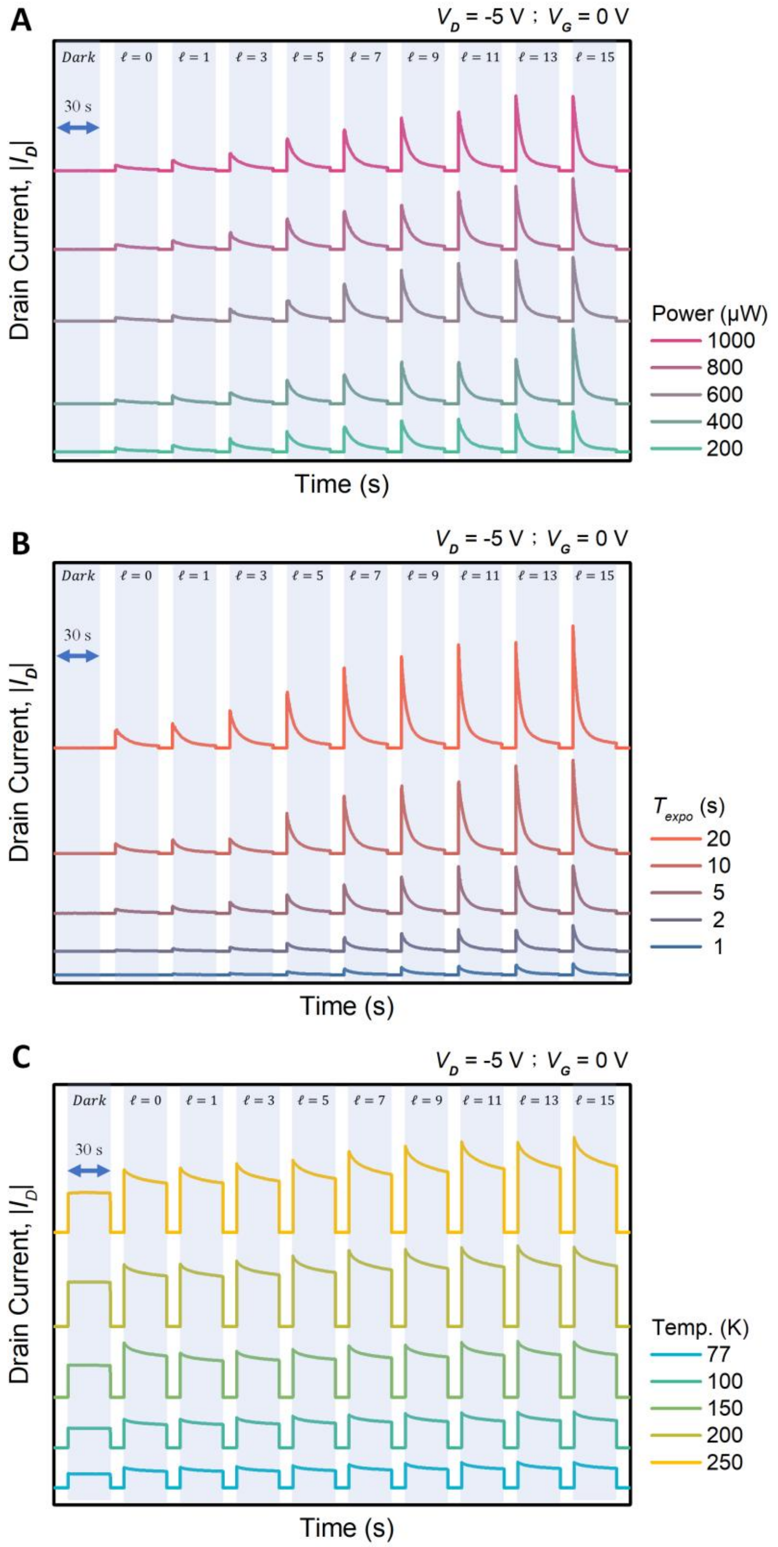

Figure S5. Dependence of current readout on power, exposure time, and temperature.
The $I_D - t$ readout results under the conditions of $V_D = -5$ V, $V_G = 0$ V, and time duration 30 seconds for **(A)** power, **(B)** exposure time, and **(C)** temperature dependence.

Section 6. Environment and temperature dependence of device hysteresis sweeping.

Figure S6A presents the device $I_D - V_G$ hysteresis sweep of the device under atmospheric and vacuum conditions, revealing a reduced hysteresis window in a vacuum environment. **Figure S6B** shows that the hysteresis window decreases as the temperature decreases, suggesting that the memory effect is attributed to artificial trap states.

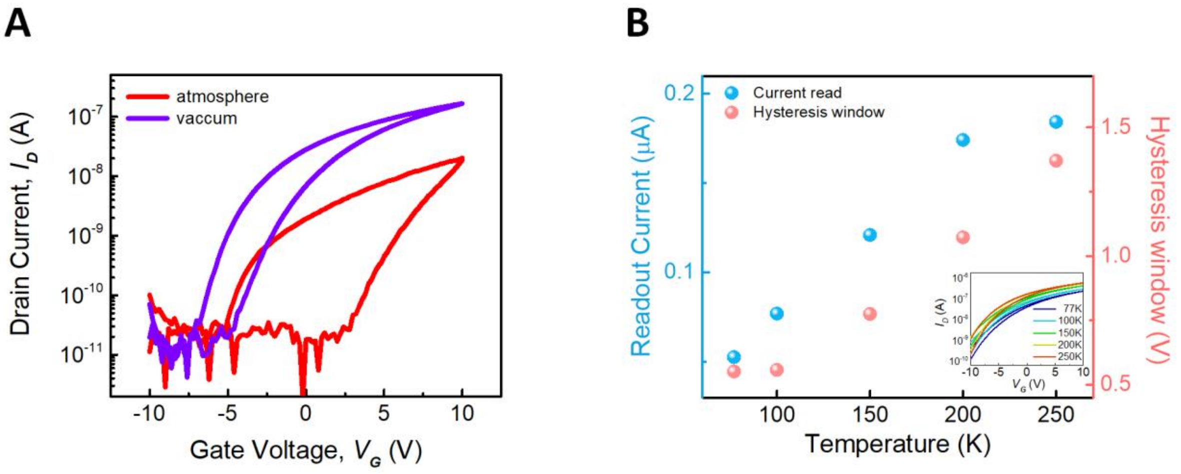

Figure S6. Environmental and temperature effects on memory characteristics.

(A) $I_D - V_G$ curve controlled by vacuum and atmosphere conditions. **(B)** The results show the device's readout current (left) and hysteresis window (right) as the temperature varies from 77K to 250K. The inset presents the raw data of the $I_D - V_G$ hysteresis sweep.

Section 7. Impact of local modulation of the effective Schottky barrier

In order to account for the potential impact of local modulation of the Schottky barrier by the spatial distribution of the OAM beam, we plotted the logarithm of the drain current versus the inverse of temperature (**Fig. S7A**) and performed a linear fit to extract the effective Schottky barrier height $[\ln(I_D) \propto \exp(q\Phi_{SB}/k_BT)]$ following optical programming (*39*).

Our analysis reveals that, although the light spot shifts laterally with increasing OAM, bringing it closer to the electrodes, the effective Schottky barrier height at the Ti/Au–TMD interface rises around 1 meV after programming (**Fig. S7B**). This finding indicates that local gating effects associated with the spatial variation of the beam slightly alter the effective Schottky barrier, thereby demonstrating that the observed memory effects are not dominated by local modifications of the metal–semiconductor interface.

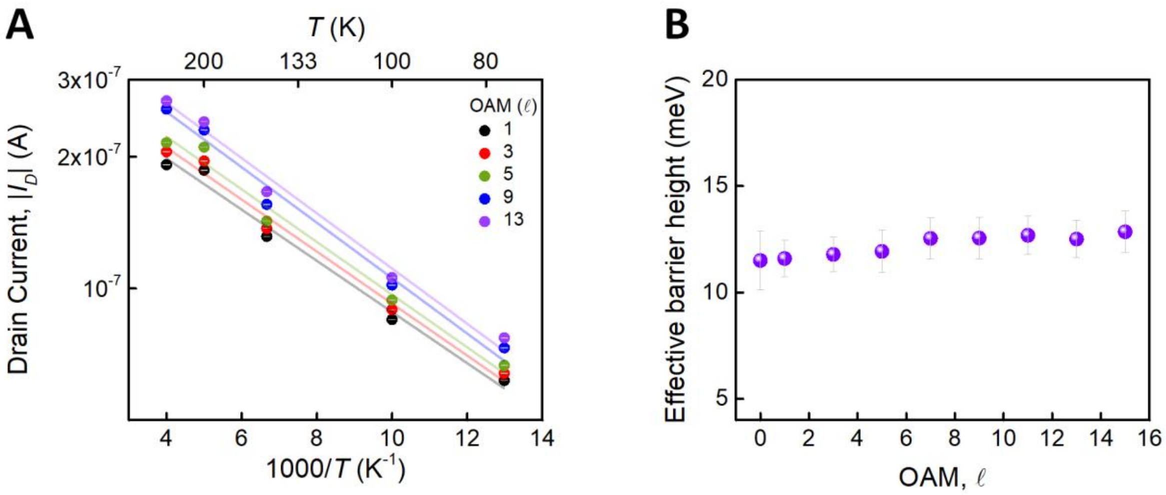

Figure S7. OAM-dependent modulation of the effective barrier height.

(A) The logarithm of the drain current versus the inverse of temperature with the programming of different order of OAM. **(B)** The extracted effective barrier height varies with the OAM light.

Section 8. Device retention time by irradiating the OAM of light.

We conducted extended endurance measurements using another device fabricated under the same oxygen plasma treatment. This device underwent a 1000-cycle test, and the results are provided in **Fig. S8A**, further supporting the reproducibility and robustness of the memory performance through the OAM of light. **Fig. S8B** illustrates the retention time of the device, defined as its ability to retain charges during continuous readout. The result indicates that the device can maintain the written state from different OAM lights for up to 100 seconds. However, after 120 seconds of readout, the current approaches the dark current readout level, losing the written state.

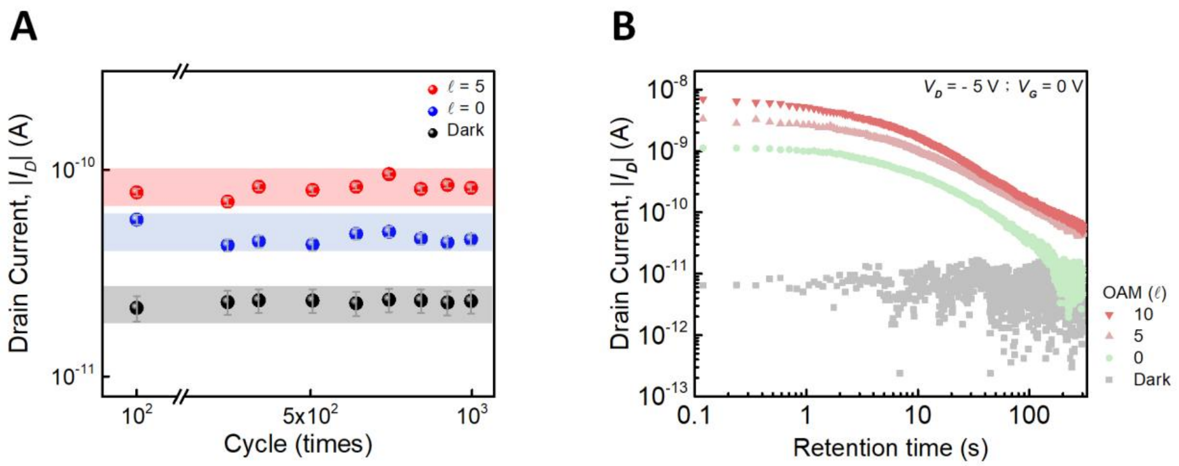

Figure S8. Extended endurance and retention performance of the memory device.

(A) The extended endurance measurements using another device fabricated under the same oxygen plasma treatment underwent a 1000-cycle test. **(B)** The retention characteristics of the memory device. The currents read under the program and erase states with V_D = -5 V and V_G = 0 V.

Section 9. Estimation of longitudinal field (E_z) strength

To estimate the strength of longitudinal fields (E_z) from the OAM of lights, we utilize equation (3) from the main text, as shown below,

$$\boldsymbol{E}(\boldsymbol{r},t) = E_0\{(\alpha\hat{\boldsymbol{x}} + \beta\hat{\boldsymbol{y}}) + \hat{\boldsymbol{z}}\frac{i}{k}[\alpha\left(\gamma\cos\phi - \frac{i\ell}{r}\sin\phi\right) + \beta(\gamma\sin\phi + \frac{i\ell}{r}\cos\phi)]\}f_{LG}, \quad \text{(S1)}$$

$$f_{LG} = \sqrt{\frac{2p!}{\pi\omega_0^2(p+|\ell|)!}}\frac{\omega_0}{\omega(z)}\left(\frac{\sqrt{2}r}{\omega(z)}\right)^{|\ell|} L_p^{|\ell|}\left[\frac{2r^2}{\omega^2(z)}\right] e^{\left[\frac{-r^2}{\omega^2(z)}\right]} \times e^{i\left[kz+\ell\phi-\omega t+\frac{kr^2}{2R(z)}-(2p+|\ell|+1)G(z)\right]}, \quad \text{(S2)}$$

$$\gamma = \left(\frac{|\ell|}{r} - \frac{2r}{\omega^2} + \frac{ikr}{R} - \frac{4r}{\omega^2}\frac{L_{p-1}^{|\ell|+1}}{L_p^{|\ell|}}\right). \quad \text{(S3)}$$

Since the incident light used in our study is linearly polarized, we simplify the calculations by setting $\alpha = 1$ and $\beta = 0$. The other parameters are defined as follows: the speed of light, $c = 3 \times 10^8$ (m/s); vacuum permittivity, $\varepsilon_0 = 8.854187817 \times 10^{-12}$ (F/m); wavelength, $\lambda = 532 \times 10^{-9}$ (m); beam waist, $\omega_0 = 10 \times 10^{-6}$ (m); and $z = 0$ (m), corresponding to the focal plane. Under these conditions, we neglect the Gouy phase (G) and the radius of curvature (R), assuming the beam radius is equal to the beam waist ($\omega = \omega_0$) in our estimation. The field amplitude (E_0) is determined by referencing the irradiating power ($P = 600$ μW) utilized during the programming procedure,

$$E_0 = \sqrt{\frac{2P}{c\epsilon_0 \iint |E(r,\phi)|^2 dA}}. \quad \text{(S4)}$$

The radius of the total irradiated area is assumed to be five times the beam waist ($r_{max} = 5\omega_0$), resulting in a total area $A = \pi r_{max}^2$. To estimate the field strength, we first integrate the squared electric field over the area A,

$$S = \int_A |E|^2 dA = \int_0^{2\pi}\int_0^{r_{max}} r|E|^2 dr d\phi. \quad \text{(S5)}$$

Finally, the area-averaged electric field amplitude within the irradiated region is calculated by the squared electric field over the entire illuminated area and then taking the square root of the average value,

$$E_{avg} = \sqrt{\frac{S}{A}}. \quad \text{(S6)}$$

This area-averaged quantity provides an effective representation of the field strength associated with the total optical energy delivered during excitation. In practice, objective lenses are used to focus the light source, making it essential to account for the lens's numerical aperture (NA). Considering the NA introduces variations in the incident angles of the focused beam. These variations result in the transverse field acquiring a z-component due to the non-zero incident angles. Additionally, the OAM-induced longitudinal field ($E_{z,longitudinal}$) generates corresponding transverse components. As a result, the total equivalent field strength ($E_{avg,total}^{\ell}$) is calculated as the summation of the equivalent transverse field ($E_{avg,transverse}^{\ell}$) and the OAM-induced longitudinal field ($E_{avg,longitudinal}^{\ell}$),

$$E_{avg,total}^{\ell} = E_{avg,transverse}^{\ell} + E_{avg,longitudinal}^{\ell}. \quad \text{(S7)}$$

In this study, a 10× objective lens with a numerical aperture (NA) of 0.25 is used. As illustrated in **Fig. 5C**, the estimated total equivalent field strength shows contributions from the transverse component (black) and the longitudinal component (red). Notably, the longitudinal field strength increases with the rising ℓ, while the transverse field strength exhibits a slight decrease.

Section 10. Examination of positive and negative OAM

The experimental results show that, for low-order OAM beams, the device exhibits a symmetric readout hysteresis window under both positive and negative OAM conditions, suggesting that the memory effect in this regime is primarily governed by the intensity distribution rather than the handedness of the helical phase. However, for higher-order OAM beams, this symmetry gradually breaks down. This asymmetry is likely amplified by residual imperfections in the optical system, particularly the sensitivity of certain optical elements, such as lenses and mirrors, to the sign of the helical phase at high OAM orders. These components may preferentially distort or attenuate either the positive or negative OAM beam, leading to unequal excitation conditions. Additionally, the increased sensitivity of the system to thermal fluctuations under repeated measurements, especially at high OAM, can introduce local variations in charge trapping near the electrodes.

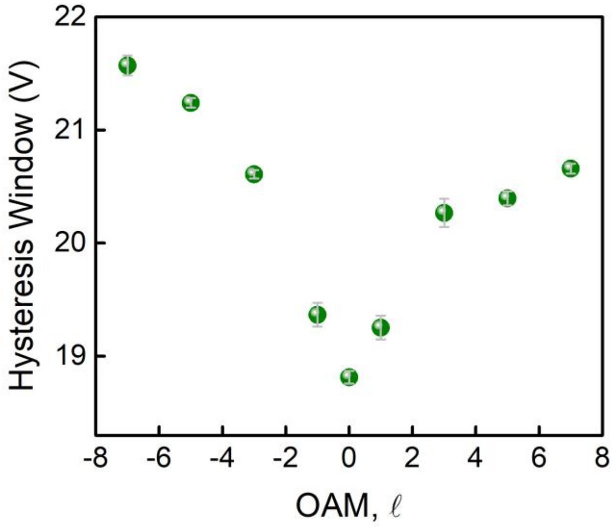

Figure S9. The readout hysteresis window through positive and negative OAM.

Section 11. Examination of OAM on other devices made of different materials.

Figure S10A indicates that the graphene-based floating-gate memory device is composed of a Si/SiO_2 substrate, a graphene channel, an h-BN that serves as an insulating layer, and a layer of MoS_2 that serves as a storage layer. The SiO_2 layer serves as the insulator for the control of the bottom gate to process the erase and write operations, where the thickness is 30 nm. The graphene, spanning between two electrodes, forms a channel with a length of 100 μm and a width of 60 μm. The h-BN layer acts as an insulator to prevent charge leakage and as a tunneling layer for the erase and write processes. Finally, the MoS_2 functions as the floating gate that stores charge. The electrical measurement results are shown in **Fig. S10C** and **S10D**. **Figure S10C** displays the $I_D - V_G$ measurements taken in a dark environment, where it can be seen that the current varies controllably with the gate voltage. Due to the unique band structure of graphene, the channel exhibits n-type or p-type behavior when the gate voltage exceeds or falls below certain values, respectively. The hysteresis window observed in the bidirectional $I_D - V_G$ readout confirms the operation of the MoS_2 floating gate. The device was written with light-carrying OAM and read with $I_D - t$, as shown in **Fig. S10D**. The black line represents the current read from the device without using light for writing, while the other lines correspond to different OAM values (ℓ). It is observed that the read current decreases as the ℓ value increases, verifying that under V_G = 0 V conditions, the device's current is in the p-type characteristic region as indicated in **Fig. S10C**.

The structure of a pentacene-based polymer memory device is composed of the Si/SiO_2 substrate, F8BT, and pentacene, as shown in **Fig. S10B**. The silicon dioxide layer acts as the bottom gate to control the erase procedure, F8BT serves as the charge storage layer, and the pentacene serves as the channel layer for electrical readout. We conducted the light-carrying OAM for the writing process of this polymer memory device and performed $I_D - V_G$ and $I_D - t$ readout of its electrical properties. The results are shown in **Fig. S10E** and **S10F**. In **Fig. S10E**, the $I_D - V_G$ readout results in a slightly increased hysteresis window as the rising ℓ is used. The black line represents the readout of the $I_D - V_G$ curve without the writing process. Similarly, the $I_D - t$ readout, as shown in **Fig. S10F**, results in different current states after the writing process with different OAM of light.

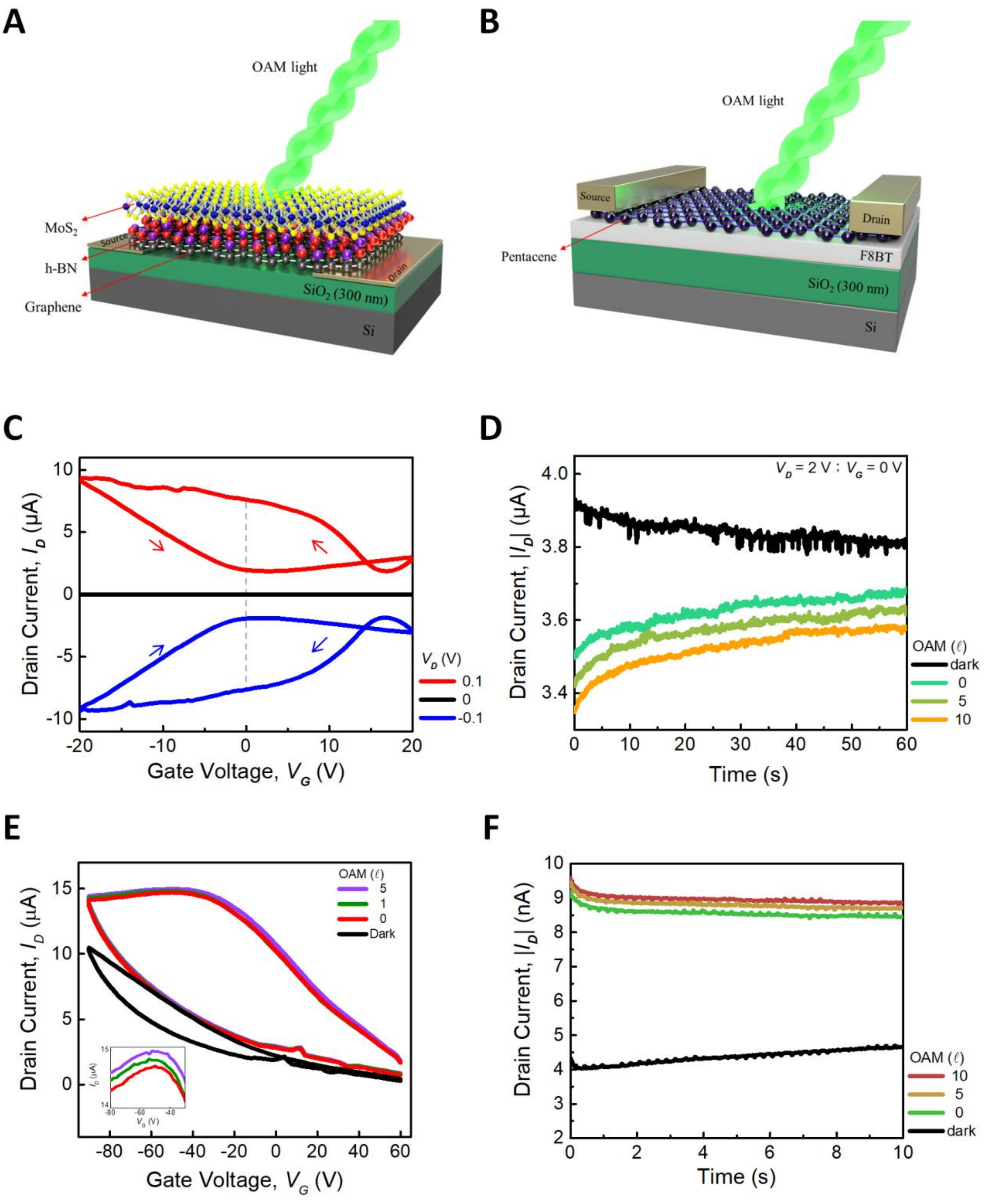

Figure S10. OAM light control of various memory devices based on different material systems.
(A) Illustrate the structure of a graphene-based floating gate memory device. **(B)** Illustrating the structure of a pentacene-based polymer material memory device. **(C)** Graphene-based floating gate memory $I_D - V_G$ curve. **(D)** The current readout by $I_D - t$ for the graphene-based floating gate memory controlled by OAM light. **(E)** Polymer-material base memory $I_D - V_G$ curve controlled by OAM light. **(F)** The current readout by $I_D - t$ for the pentacene-based floating gate memory controlled by OAM light.

Section 12. Device performance metrics

Table S1. Device performance metrics of TMD-based charge trapping photomemory devices

Ref.	Active channel	Storage medium	Maximum I_{on}/I_{off}	Retention (s)	Endurance (cycles)	Erase operation	Operation speed(s)	Programming Operation	Operation speed(s)	Laser's degree of freedom
(*47*)	MoS_2	AuNP	$> 10^7$	$>10^4$	> 200	Gate pulse 100 V	1	Gate or Laser pulse	1	Pulse intensity
(*48*)	MoS_2	Artificial trap sites at MoS_2/SiO_2 substrate	4700	10^4	N/A	Gate pulse 80 V	1	Laser pulse	0.1	Multiple pulses
(*49*)	MoS_2	PbS	600	10^4	2000	Gate pulse 40 V	0.1	Laser pulse	5	Multiple pulses
(*50*)	$MoS_{2x}Se_{2(1-x)}$	Trap states induced by defects in active channel	10^8	10^4	> 600	Gate pulse -80 V & Laser pulse	1	Gate pulse 80 V	1	N/A
(*51*)	WSe_2	Mid-gap states of h-BN	10^6	4.5×10^4	200	Gate pulse 50 V & Laser pulse	2	Gate pulse -20 V with Laser pulse	0.5	Wavelength
This work	MoS_2	Artificial trap sites at MoS_2/SiO_2 substrate	10^2	120	> 1000	Gate pulse 40 V	3	Laser pulse	2	Pulse intensity and **OAM (ℓ)**
